# Effects of combinatorial hurdles on a non-alcoholic beer matrix challenged with *Salmonella* Javiana*, Escherichia coli, Listeria monocytogenes, Pseudomonas aeruginosa, and Bacillus cereus*

**DOI:** 10.3389/fmicb.2026.1835393

**Published:** 2026-05-18

**Authors:** Andrew Maust, Karina Desiree, Peter M. Rubinelli, Sungil Ferreira, Jennifer C. Acuff, Scott Lafontaine

**Affiliations:** Department of Food Science, University of Arkansas, Fayetteville, AR, United States

**Keywords:** anti-microbial, challenge study, food safety, hurdle technology, non-alcoholic beer

## Abstract

Growing consumer interest in reducing alcohol consumption is prompting beverage producers to develop non-alcoholic versions of traditional products; however, the removal of ethanol also removes an important food safety hurdle. This study evaluated whether the remaining intrinsic hurdles of pH (5.0 or 4.2), carbonation (0, 0.75, or 1.5 volumes CO₂), and antimicrobial compounds (none, 10 ppm iso-*α*-acids, or 100 ppm potassium sorbate) were sufficient to control foodborne pathogens in a non-alcoholic beer matrix. A factorial combination of these variables produced 18 treatments, with 2 additional alternative treatments representing kettle souring and chitosan addition. A five-strain bacterial cocktail containing *Salmonella enterica* serovar Javiana, *Escherichia coli* O157: H7*, Listeria monocytogenes* 4b, *Pseudomonas aeruginosa*, and *Bacillus cereus* (1 × 10^4.8 CFU/mL each) was inoculated into 10.8 oz. of a model non-alcoholic beer and monitored over 60 days. Samples were analyzed in triplicate on days 1, 7, 14, 28, and 60 using selective and differential plating methods. pH was identified as a critical factor for pathogen control, both directly and through the enhancement of other hurdles. Carbonation at 1.50 volumes effectively controlled *S.* Javiana and *E. coli* bacteria, whereas reduced carbonation levels supported their growth and survival. Hop acids contributed to additional control of Gram-positive bacteria. Several treatments reduced pathogen populations to below the limit of detection (<10 CFU/mL), corresponding to reductions greater than 3 log units, whereas high-pH treatments lacking additional hurdles supported pathogen growth and survival. These findings demonstrate that recipe design strongly influences the intrinsic antimicrobial stability of non-alcoholic beer and provides practical guidance for producers and process authorities evaluating microbial risk and processing needs in this emerging category.

## 1 Introduction

Beer and similar alcoholic beverages, such as hard seltzers, have historically been seen as safe from pathogenic bacteria, tracing their protection to several chemical hurdles, such as alcohol, low pH, carbonation, and the presence of antimicrobial compounds, such as hop acids (Menz et al., 2009). Recently, however, non-alcoholic beer (NAB) has been rising in popularity due to increased consumer health consciousness and warnings about alcohol consumption (Davis, 2023; Office of the Surgeon General, 2025). When intrinsic hurdles are removed, such as alcohol, the food safety of these emerging beverages needs to be re-evaluated to understand the degree to which remaining hurdles provide control of foodborne pathogens.

A critical intrinsic factor that can be manipulated for food safety is pH. Common alcoholic beer pH typically falls between 3.4 and 4.8, with Menz et al. (2009) finding the average level between 3.9 and 4.3. Best practices for NAB production recommend a pH of 4.2 or less; however, surveys of commercial products have revealed products as high as pH 5.0 (Lafontaine et al., 2020; Britton and Hill, 2025). The FDA uses pH 4.6 as the legal line between high acid (≤4.6) and low acid (>4.6) foods, as this is the minimum value needed to inhibit the growth and toxin production of *Clostridium botulinum* (21 C.F.R. § 114; FDA, 2012). *C. botulinum* is of particular concern to public health due to its thermoduric tolerance, low infective dose, and life-threatening symptoms (FDA, 2012). However, other thermoduric spore formers like *Bacillus cereus* have been discovered growing in even lower pH beer environments (pH 4.3), with these select strains exhibiting acid adaptation (Wang et al., 2017). Vegetative pathogens also display the ability to adapt to acidic conditions, presenting a potential risk at typical beer pH’s. These include *Listeria monocytogenes, Salmonella* spp., and *Escherichia coli* O157:H7, which can grow at a pH of 4.4, 4.1, and 4.0, respectively, in the absence of other hurdles, and with evidence of the ability to present acid tolerance responses upon exposure to sub-lethal acidic environments (Smith et al., 2013; Usaga et al., 2014; Gavriil et al., 2024; Rachon et al., 2024).

Carbonation has traditionally been held as incompatible with the growth of pathogens. However, most research has focused on sparkling water or carbonated soft drinks, which would present a matrix with either limited nutrients or significantly lowered pH. Late 19th and early 20th century testing of still versus sparkling waters showed that carbonation led to decreases in bacterial populations over storage time (Koser and Skinner, 1922; Witter et al., 1958). The conditions of carbonation for these waters and soft drinks are also at volumes higher than typically found in the brewing industry (≥3 vs. 2.3 volumes, respectively) (Buiatti, 2009; Azeredo et al., 2016). Work by Insalata (1952) found that a 1.7 log reduction of *E. coli* occurred in a sterilized sucrose solution at 2.7 volumes of CO_2_ after 6 days. Experiments by Witter et al. (1958), found that populations of *E. coli* in a 10 °Brix solution at pH 4.5 started decreasing when carbonation levels were increased from two to three volumes. Prior work in non-alcoholic beer identified that carbonation helped to prevent the growth of pathogens over un-carbonated samples; however, a critical limit was not established for this effect by Rachon et al. (2024). From a regulatory perspective, the FDA grants exemptions to carbonated beverages from following the full acidified foods process scheduling that is outlined in 21 C.F.R. §114. This is likely based on the earlier work conducted in carbonated soft drinks, but may need to be re-validated with challenge testing at higher pH and lower carbonation volumes to match the profile of NAB.

Hops have a long history of being recognized for their anti-microbial properties, conveying preservation to beer (Moir, 2000; Srinivasan et al., 2004; Bocquet et al., 2018). At moderate usage rates (< 20 International Bitterness Units [BU]), there is a proven efficacy against Gram-positive bacteria of both pathogenic and spoilage classification (Menz et al., 2010; Schurr et al., 2015). Several pathogens that have been shown to be controlled by hop acids in a NAB-like matrix include *L. monocytogenes*, *Staphylococcus aureus*, and spore formers, such as *B. cereus* (Menz et al., 2010). Gram-negative pathogens, such as *E. coli* and *Salmonella* spp., are not typically inhibited by hop acids, owing to the protection to serum phosphatide production in their cell walls (Sacks and Humphreys, 1951; Menz et al., 2010).

Chemical preservatives are commonly added to beverage products to help prevent the growth of yeasts and molds, which can be hard to control with other intrinsic hurdles or thermal processing (Wareing and Davenport, 2004; Stratford et al., 2020). A common preservative class is weak acid antimicrobials, such as benzoic acid or sorbic acid, which rely on passive diffusion of undissociated acids across bacterial cell membranes, before dissociating in the cytoplasm, lowering intracellular pH (Lambert and Statford, 1999). As the action mechanism for these preservatives is the concentration of undissociated acids, pH is a critical co-factor for their efficacy, with sorbic acid active over a greater range of moderately acidic pH levels than benzoic acid (Lambert and Statford, 1999). The addition of these preservatives is mainly serves as a fungistatic agent, although some bacteriostatic activity has been shown against *B. cereus*, *E. coli*, *Psuedomonas aeruginosa*, *L. monocytogenes*, and *Salmonella* spp., although these minimum inhibitory concentration (MIC) values are higher than those needed to control fungal species, and acceptable usage rates should not rise to a level high enough to impact taste (LaRocco and Martin, 1981; Eklund, 1983; El-Shenawy and Marth, 1988; Stopforth et al., 2005). Prior research in wine has placed the sensory threshold for sorbic acid between 240 and 400 ppm (Tercelj and Adamic, 1965; Tromp and Agenbach, 1981).

Current best practices for non-alcoholic beer production involve tunnel or batch pasteurization of final packaging for destruction of vegetative pathogens, combined with a pH below 4.2 to prevent the sporulation of pathogens such as *B. cereus* and *C. botulinum* (Rachon et al., 2024; Britton and Hill, 2025). However, producers may have concerns that thermal pasteurization could lead to heat damage to the flavor of their products, as well as carry prohibitive capital costs to purchase a tunnel pasteurizer (Cao et al., 2011; Wray, 2025). This may lead them to explore non-thermal preservation techniques, such as chemical preservatives. Additionally, thermal pasteurization alone will not provide adequate protection for draft products, which may be exposed to post-lethality contamination during their tapping and serving (Quain, 2021; Roselli et al., 2026).

When producing shelf-stable products without a terminal heat treatment (cold-fill-hold) and relying on their formulation and chemical preservatives *in lieu* of a thermal process, the FDA expects scientific validation of safety, typically via a product-specific challenge study or by citing adequately similar peer-reviewed studies, so that the scheduled process is supported by sound data. This is standard practice for acidified foods under 21 CFR 114 and FSMA Preventive Controls for Human Foods Rule. A second option is the use of publicly available data that emulate the formulation (acid type and concentration), equilibrium pH, and storage conditions to demonstrate an adequate pathogen lethality (many studies target ≥5-log reduction for acid/acidified foods processed by cold-fill-hold). Looking at cold-fill-hold, a Process Authority can establish scheduled processes grounded in published challenge-study conditions, for example: acetic acid as the acidulant with a maximum equilibrium pH ≤ 3.30 plus a defined hold-time/temperature (e.g., ~6 days at 10 °C or ~35 h at 25 °C) to ensure ≥ 5-log reduction (Breidt et al., 2007); or formulations with 2.5% acetic acid at max pH ≤ 3.50; or 2.5% acetic acid + 0.1% benzoic acid at max pH ≤ 3.80—with hold conditions matched to the study (Breidt et al., 2013). These parameters are used widely for pickles, sauces, and dressings, and could be adapted, with equivalency justified, to non-alcoholic beverages when a thermal step is impractical; however, sensory constraints limit acetic acid in beverages. Altogether, this underscores the need for peer-reviewed, formulation-specific studies to support safe, efficient, and high-quality production of non-alcoholic beverages using cold-fill-hold or at lower pasteurization conditions.

Previous studies on the safety of NAB have raised concerns about the possibility of pathogenic growth, especially *Salmonella* spp. and *E. coli* (Menz et al., 2010; Çobo et al., 2023; Frierson et al., 2026). However, the tested matrices of these studies were not fully representative of the NAB environment, leaving out carbonation, which was established as a critical intrinsic factor limiting pathogen growth by Rachon et al. (2024). This study aims to investigate chemical hurdles in a representative combinatorial design, providing insight into how formulation design may impact the overall safety of NAB against common food pathogens. This can provide brewers and process authorities with greater insight into how unique NAB matrices, with varying pH’s, carbonation levels, and antimicrobial constituents, convey protection against pathogens.

## 2 Materials and methods

### 2.1 Experimental design and NAB preparation

**Figure 1:**
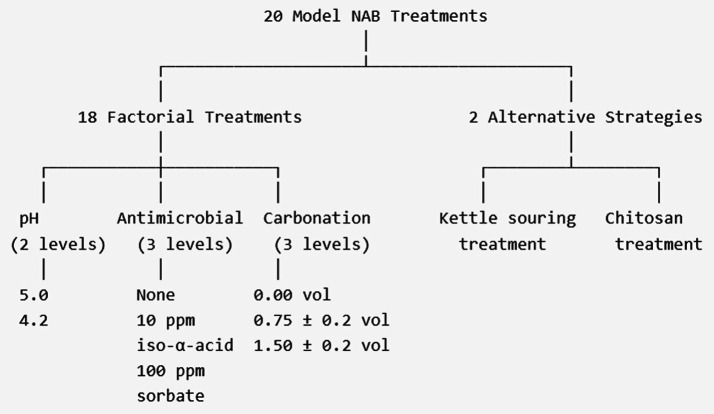
Experimental design was used to generate 20 model non-alcoholic beer treatments. Eighteen treatments were prepared as a full factorial combination of two pH levels (5.0 and 4.2), three antimicrobial conditions (none, 10 ppm iso-α-acid, or 100 ppm potassium sorbate), and three carbonation levels (0.00, 0.75 ± 0.2, or 1.50 ± 0.2 volumes CO₂). Two additional treatments represented alternative protection strategies: kettle souring and chitosan addition.

A total of 20 unique treatment conditions were crafted to create 20 different model non-alcoholic beers (Figure 1; Supplementary Table S1). The first 18 were a factorial combination of two levels of pH (5.0 or 4.2), three levels of antimicrobial (none, 10 ppm iso-*α*-acid (Hopsteiner, New York, NY, USA), or 100 ppm potassium sorbate (North Mountain Supply, Mildred, PA, USA)), and 3 levels of carbonation (0.00 volumes, 0.75 (± 0.2) volumes, or 1.50 (± 0.2) volumes). The remaining two treatments represent alternative NAB protection strategies, kettle souring, and addition of chitosan (Chiber Chinova Bioworks, Fredericton, New Brunswick, Canada).

The NAB matrices were prepared by dissolving Pilsen Dry Malt Extract (DME) (LD Carlson, Briess Malting and Ingredients, Chilton, WI) in 65 °C water to a final concentration of 6 °P (± 0.2 °P), and using citric acid to adjust pH down to 5.0 (± 0.03). The antimicrobials (minus the chitosan treatment) were introduced at this stage to ensure consistent dosing across pH treatments. Two-gallon stainless steel kegs (G4 kegs, Virginia Beach, VA) were filled with 7 L of the pH 5.0 treatments. Then, more citric acid was added to the DME solution to lower the pH to 4.2 (± 0.03) before filling the remaining kegs. Kegs were submerged in boiling water for 20 min, receiving over 4.5 million pasteurization units (PU) (Carl, 1995). This level aimed to eliminate all vegetative organisms, although it would not destroy spores. Kegs were stored under refrigerated conditions until the time of filling. Carbonation was applied by setting head pressure to 8 psi and 15 psi for the 0.75 vol and 1.50 vol treatments, respectively, over a 3-day period. Carbonation was checked by filling test bottles and evaluating them with an Anton Paar C-box with PFD filling attachment (Graz, Austria).

The kettle sour was prepared by taking 6 °P DME and pitching with a *Lactiplantibacillus plantarum* culture (Fermentis, Lille, France). The temperature was held at 35 °C for 36 h until the pH dropped below 3.4, before bringing the solution up to a boil to inactivate the bacteria. The kettle sour was kegged and subjected to the same pasteurization treatment.

The commercial chitosan blend (Chinova Bioworks, Fredericton, New Brunswick, Canada) was dosed at the time of filling to 0.2% at pH 5.0, no antimicrobial, amd 0.00 vol carbonation treatment base.

### 2.2 Inoculum and sample preparation

Isolates of five pathogens were prepared first as individual inocula in this study: *Salmonella enterica* subsp*. enterica* ser. Javiana (BAA-1593), *Escherichia coli* O157: H7 (ATCC 1357), *Listeria monocytogenes* Scott A (4b, ATCC 43257, clinical isolate from Institute for Food Safety and Health, Illinois Institute of Technology, Bedford Park, IL), *Pseudomonas aeruginosa* (ATCC 10145), and *Bacillus cereus* (ATCC 14579).

The cultures were maintained at −80 °C on protectant beads in tryptic soy broth (TSB, Becton, Dickinson and Company, Sparks, MD 21152 USA) with glycerol (Microbank™, Pro-Lab Diagnostics, Ontario, Canada). A single bead of each isolate was removed and added to individual tubes of 10 mL sterile TSB. Cultures were incubated overnight at 37 °C for 24 h. From the overnight culture, an isolation streak of each culture was performed on tryptic soy agar (TSA, Becton, Dickinson and Company, Sparks, MD 21152 USA) and incubated at 37 °C for 24 h. A single colony of each culture was removed and added to 10 mL of sterile TSB for an overnight culture incubated at 37 °C for 24 h. The overnight culture was centrifuged at 8000 x g for 5 min (Eppendorf 22,331, Hamburg, Germany). The supernatant was gently discarded and the cells from the pellet were washed twice by resuspending them in 10 mL of sterile 0.1% peptone (Hardy Diagnostic, Santa Maria, CA). The suspension was quantified using the Petroff-Hausser (PH) counting chamber (Hausser Scientific, PA 19044, USA), as directed by the manufacturer. Once quantified, the concentration of each culture was adjusted to 1 × 10^8^ CFU/mL using sterile TSB. Equal volumes of the five cultures were transferred to a single tube to serve as a cocktail.

A cycloheximide (Sigma-Aldrich, Inc., MO 63178, USA) stock solution (2,310 ppm) was prepared by dissolving the powder in water with continuous stirring. This concentration was selected to yield a final concentration of 7 ppm in each bottle, which has been shown to effectively suppress contaminants from the brewing environment (e.g., brewer’s yeast) without affecting the growth of the inoculated pathogens (Ha et al., 1995).

### 2.3 Bottling

Prior to filling, 12 oz amber glass beer bottles (The Cary Company, Addison, IL) were sterilized by autoclaving. Bottle caps (North Mountain Supply, Mildred, PA) and Beer Gun (Blichmann Engineering, Lafayette, IN) were soaked in a quaternary ammonium compound (QUAT; Bacdown® Detergent Disinfectant, Decon Labs, King of Prussia, PA) sanitizer for 20 min, rinsed using sterile distilled water, and dried within a biosafety cabinet. During filling, approximately 320 mL (10.8 oz) of the model beer treatment was dispensed into each bottle, followed by the addition of 1 mL of bacterial cocktail and 1 mL of cycloheximide stock solution. Non-carbonated samples were dispensed under nitrogen, and bottles were not purged of oxygen before filling. Carbonated samples were dispensed using CO_2_ and had bottles purged with CO_2_ for 5 s to reduce package oxygen. Negative controls were created for each treatment by filling bottles with the same volume of model beer with the addition of cycloheximide, but without the inoculum. Bottles were securely capped and hermetically sealed with wax before storing at room temperature (25 °C).

### 2.4 Sample enumeration

Starting bacterial concentrations were calculated at 4.8 log CFU/mL for each bacterium based on the dilution of the bacterial cocktail in 322 mL and verification with pre-trial testing (results not shown). Samples were enumerated on day 1, 7, 14, 28, and 60 after filling. On sampling day, bottles were aseptically opened and stirred using a sterile serological pipette to mix their contents before drawing 1.0 mL out to make serial dilutions using 0.1% peptone (Hardy diagnostic, Santa Maria, CA). Samples dilutions were plated on CHROMagar™ *Salmonella*, CHROMagar™ *B. cereus*, CHROMagar™ *Listeria*, CHROMagar™ *Pseudomonas*, and CHROMagar™ STEC (CHROMagar™, Saint Denis, France) to enumerate each pathogen population. Negative controls were sampled on days 1 and 7 to confirm the absence of microbial growth other than the inoculated organisms. Plates were incubated at 37 °C for 48 h before being counted. The experiment was conducted in triplicate using three sets of independently prepared cultures.

### 2.5 Beer chemistry

Original gravity, real extract (RE), and pH were measured using an Anton Paar DMA 4501 benchtop densitometer with Alcolyzer 3001and pH probe 1101 attachments (Graz, Austria). Free amino nitrogen (FAN) was measured colorimetrically based on the method published by Schmitt and Budde (2010), with the analysis conducted in 96-well plates and analyzed by a Synergy HTX plate reader (Agilent, Santa Clara, CA, USA).

### 2.6 Statistical analysis

Statistical analyses (e.g., ANOVA with Post-Hoc Tukey Grouping) as well as some graphical analyses were performed with Excel for Microsoft 365 (Microsoft, Redmond, WA, USA) using XLSTAT Premium 2021.3.1 (Addinsoft, NY, USA) as well as R version 4.5.2 (R Foundation for Statistical Computing, Vienna, Austria) using the Agricolae package (Felipe de Mendiburu, Lima, Peru).

## 3 Results

### 3.1 Microbial challenge results at PH 5.0

**Figure 2:**
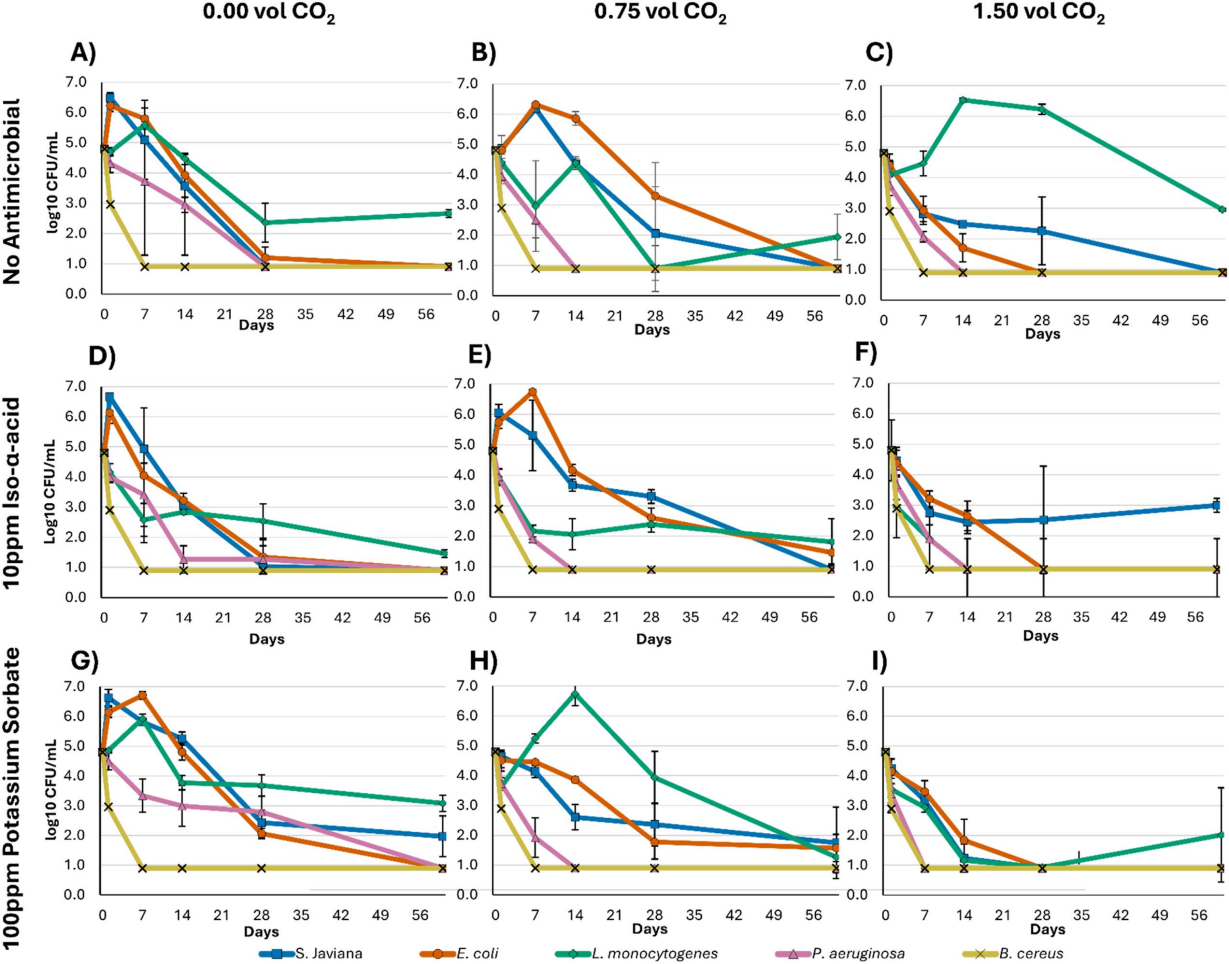
**(A–I)** Shows log-transformed microbial counts (log CFU/mL)* measured at 0, 1, 7, 14, 28, and 60 days for *Salmonella* Javiana, *Escherichia coli*, *Listeria monocytogenes*, *Pseudomonas aeruginosa*, and *Bacillus cereus* in model non-alcoholic beer treatments at pH 5.0. Treatments were as follows: **(A)** 0.00 vol CO₂, no antimicrobial; **(B)** 0.75 vol CO₂, no antimicrobial; **(C)** 1.50 vol CO₂, no antimicrobial; **(D)** 0.00 vol CO₂, 10 ppm iso-α-acid; **(E)** 0.75 vol CO₂, 10 ppm iso-α-acid; **(F)** 1.50 vol CO₂, 10 ppm iso-α-acid; **(G)** 0.00 vol CO₂, 100 ppm potassium sorbate; **(H)** 0.75 vol CO₂, 100 ppm potassium sorbate; and **(I)** 1.50 vol CO₂, 100 ppm potassium sorbate. *Limit of detection was <3 CFU/mL at day 1 (graphically represented as 2.9 CFU/mL) and <1 CFU/mL at days 7, 14, 28, and 60 (graphically represented as 0.9 CFU/mL).

At pH 5.0, the effects of carbonation level and antimicrobial treatment on pathogen survival were evaluated across the nine treatment combinations (Figure 2; Supplementary Tables S2–S7). These treatments included three carbonation levels (0.00, 0.75, and 1.50 vol CO₂) combined with either no antimicrobial, 10 ppm iso-*α*-acid, or 100 ppm potassium sorbate. This pH was chosen because it is representative of high pH products observed in the market (Lafontaine et al., 2020) and also reflects a plausible risk condition if wort were packaged without sufficient acidification, as wort commonly occurs near this pH. Thus, these treatments provide a useful model for microbial behavior under conditions relevant to real-world product safety concerns.

*Salmonella* Javiana populations grew in all the treatments with 0.00 vol carbonation, as well as the 0.75 vol carbonation samples with no antimicrobial and 10 ppm iso-*α*-acids (Figures 2A,B,D,E,G; Supplementary Table S2). The samples with 1.50 vol carbonation, as well as 0.75 vol carbonation and 100 ppm potassium sorbate, prevented growth and showed a decline of *Salmonella* Javiana populations over the length of the study (Figures 2C,F,H,I). However, over 2-log CFU/mL were still recoverable at 28 days, with the exception of the 1.50 vol carbonation and 100 ppm potassium sorbate treatment (Figure 2I).

*E. coli* showed similar trends, with growth occurring in samples at 0.00 vol carbonation, as well as the 0.75 vol carbonation samples with no antimicrobial and 10 ppm iso-*α*-acids (Figures 2A,B,D,E,G; Supplementary Table S3). Carbonating to 1.50 vol of carbonation reduced *E. coli* populations to the limit of detection (<1 log CFU/mL) in all antimicrobial treatments (Figures 2C,F,I), while 0.75 vol carbonation and 100 ppm potassium sorbate inhibited growth but did not reduce the population as effectively as the higher carbonation level (Figure 2H).

*Listeria monocytogenes* survived well and grew at pH 5.0. After an initial population decline in no antimicrobial and 100 ppm potassium sorbate treatments, enumerated populations indicated growth over storage (Figures 2A–C,G,H; Supplementary Table S4). The 1.50 vol carbonation and 100 ppm potassium sorbate treatment generally showed a decline in *L. monocytogenes*; however, between days 28 and 60, there was a population increase in one replicate, with the populations in other replications remaining at the limit of detection (<1 log CFU/mL; Figure 2I). The treatments with 10 ppm iso-*α*-acids and either 0.00 or 0.75 vol carbonation prevented growth and showed steady decline over the sampling period (Figures 2D,E). The treatment with 1.50 vol carbonation and 10 ppm iso-*α*-acids had the strongest inhibition, reaching the limit of detection by day 14 (Figure 2F).

*Pseudomonas aeruginosa* was inhibited from growth in all treatments at pH 5.0, reaching the limit of detection by day 60 (<1 log CFU/mL). However, it exhibited the greatest survival rate in treatments with 0.00 vol carbonation (Figures 2A,D,G; Supplementary Table S5), showing only a 2-log reduction in the 0.00 vol carbonation and 100 ppm potassium sorbate treatment by day 28 (Figure 2G).

*Bacillus cereus* was also inhibited in all treatments at pH 5.0, with enumerations at or below the limit of detection for all timepoints (≤ 1 log CFU/mL; Supplementary Table S6). Pre-trial work (not shown) confirmed that *B. cereus* was recoverable at timepoints 1 and 7 as an individual inoculum in a NAB matrix at pH 5.0.

### 3.2 Microbial challenge results AT pH 4.2

**Figure 3:**
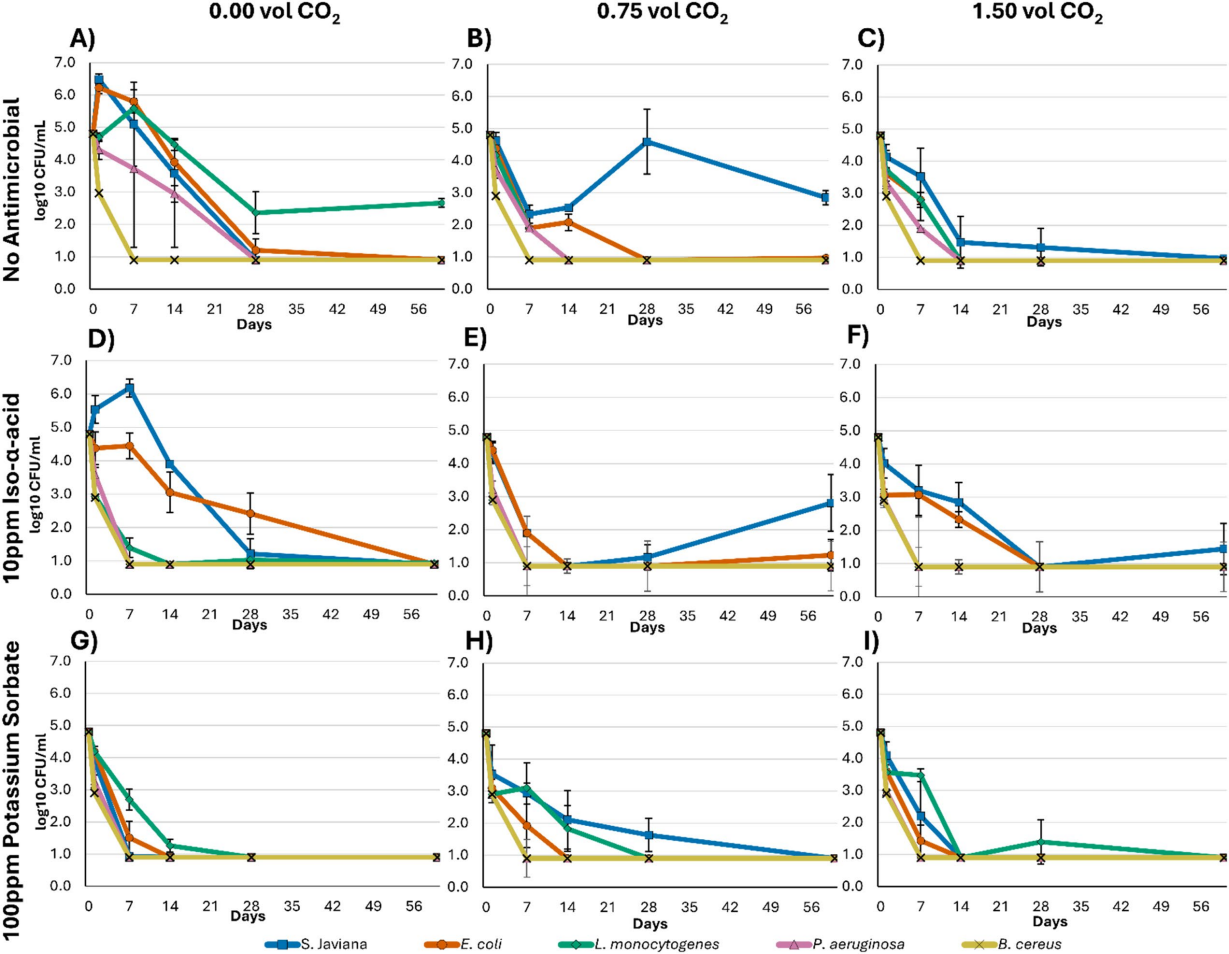
**(A–I)** Shows log-transformed microbial counts (log CFU/mL)* measured at 0, 1, 7, 14, 28, and 60 days for *Salmonella* Javiana, *Escherichia coli*, *Listeria monocytogenes*, *Pseudomonas aeruginosa*, and *Bacillus cereus* in model non-alcoholic beer treatments at pH 4.2. Treatments were as follows: **(A)** 0.00 vol CO₂, no antimicrobial; **(B)** 0.75 vol CO₂, no antimicrobial; **(C)** 1.50 vol CO₂, no antimicrobial; **(D)** 0.00 vol CO₂, 10 ppm iso-α-acid; **(E)** 0.75 vol CO₂, 10 ppm iso-α-acid; **(F)** 1.50 vol CO₂, 10 ppm iso-α-acid; **(G)** 0.00 vol CO₂, 100 ppm potassium sorbate; **(H)** 0.75 vol CO₂, 100 ppm potassium sorbate; and **(I)** 1.50 vol CO₂, 100 ppm potassium sorbate. *Limit of detection was <3 CFU/mL at day 1 (graphically represented as 2.9 CFU/mL) and <1 CFU/mL at days 7, 14, 28, and 60 (graphically represented as 0.9 CFU/mL).

At pH 4.2, which reflects the current recommended pH threshold for microbial risk reduction in non-alcoholic beer, microbial survival was evaluated across the nine treatment combinations (Figure 3; Supplementary Tables S2–S7). These treatments included three carbonation levels (0.00, 0.75, and 1.50 vol CO₂) combined with either no antimicrobial, 10 ppm iso-*α*-acid, or 100 ppm potassium sorbate. As pH 4.2 represents the presently recommended acidification target for improving product safety, these data are especially relevant for evaluating how additional hurdle technologies may further enhance microbial control during storage.

In pH 4.2 media, *S.* Javiana was still able to grow in non-carbonated samples with either no antimicrobial or 10 ppm iso-*α*-acids (Figures 3A,D; Supplementary Table S2). When 0.75 vol of carbonation was added, populations initially declined for the no antimicrobial and 10 ppm iso-α-acids treatments but began significantly increasing in population by day 28 and 60, respectively (Figures 3B,E). Further carbonation increases to 1.50 vol resulted in a reduction to the limit of detection in the no antimicrobial treatment (<1 log CFU/mL; Figure 3C), but the 10 ppm iso-α-acids treatment did see one replicate beginning to grow (Figure 3F). The potassium sorbate treatments inhibited growth and reduced *S*. Javiana to the limit of detection by day 60 across all carbonation levels (Figures 2G,H,I), although 0.75 vol carbonation presented the slowest reduction speed (Figure 2H).

*E. coli* presented weaker growth capabilities than the *S.* Javiana in the lower pH, only showing significant growth in the 0.00 vol carbonation and no antimicrobial treatment (Figure 3A; Supplementary Table S3). Growth was inhibited, but inactivation was slow, for the 0.75 vol carbonation and no antimicrobial treatment, and the 0.00 vol and 0.75 vol 10 ppm iso-α-acids treatments (Figures 3B,D,E). At day 60, enumeration above the limit of detection occurred for some replicates of 0.75 vol carbonation treatments with no antimicrobial and 10 ppm iso-α-acids, but this is not significantly differentiated from day 28 results below the limit of detection (<1 log CFU/mL; Figures 2B,E). Finally, the 1.50 vol carbonation treatments of both no antimicrobial and 10 ppm iso-α-acids, as well as all 100 ppm potassium sorbate treatments, inhibited growth and reduced populations to the limit of detection (Figures 3C,F,G–I).

*L. monocytogenes* was inhibited from significant growth at this pH level in all treatments (Supplementary Table S4), although the inactivation rates differ. The carbonated 10 ppm iso-α-acids treatments showed the fastest reduction, reaching the limit of detection at day 1 (<3 log CFU/mL) and staying below the limit of detection for the remainder of the trial (<1 log CFU/mL; Figures 3E,F).

*Pseudomonas aeruginosa* and *B. cereus* also seemingly struggled to grow in the lower pH environment (Supplementary Tables S5, S6), with all treatments having concentrations reduced below the limit of detection by days 14 and 1, respectively (<1 log CFU/mL and <3 log CFU/mL, respectively).

### 3.3 Microbial challenge results for alternative control strategies

**Figure 4:**
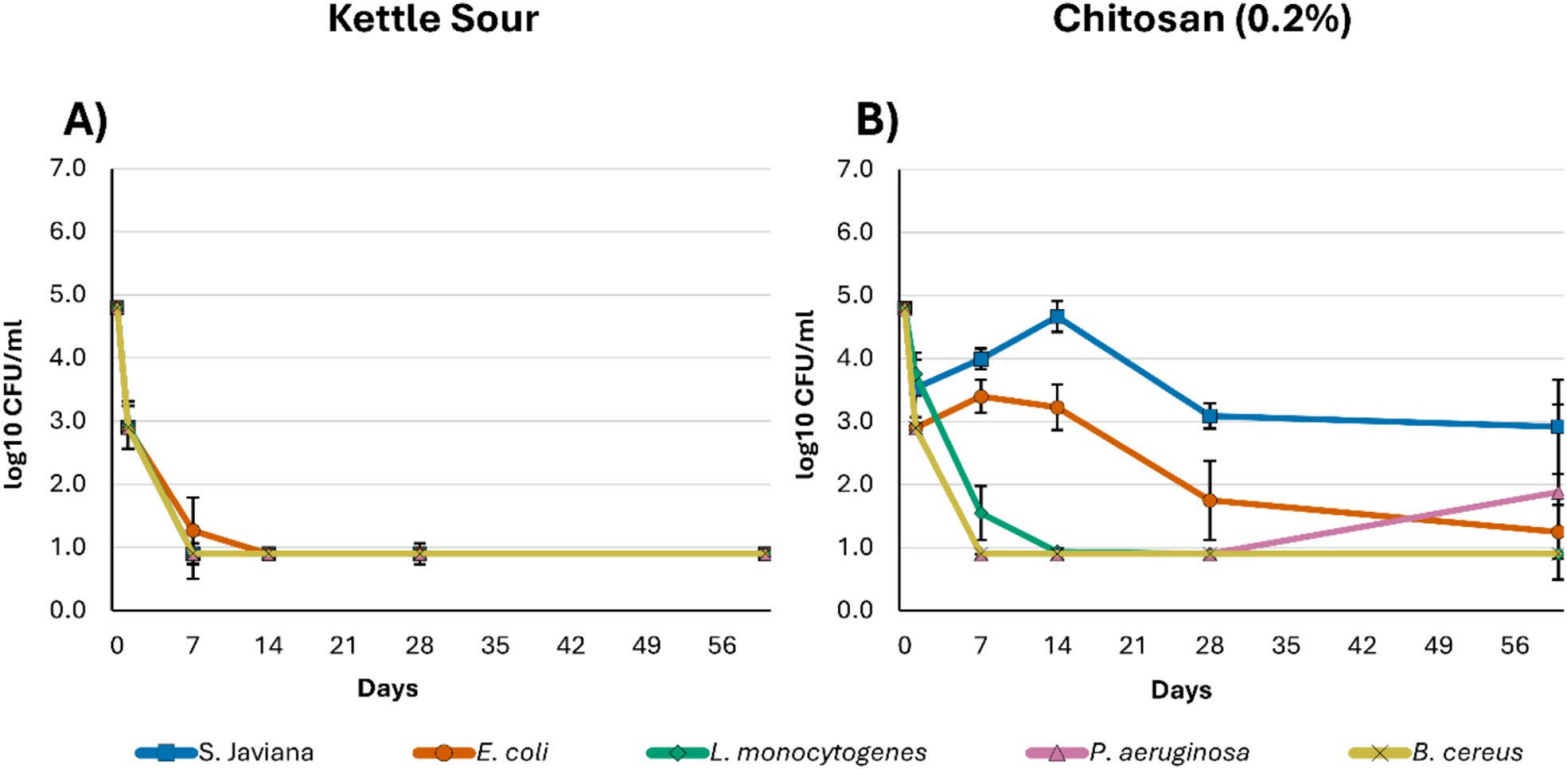
Log-transformed microbial counts (log CFU/mL)* measured at 0, 1, 7, 14, 28, and 60 days for *Salmonella* Javiana, *Escherichia coli*, *Listeria monocytogenes*, *Pseudomonas aeruginosa*, and *Bacillus cereus* in model non-alcoholic beer treatments representing two alternative protection strategies: **(A)** Kettle souring (pH 3.3, 0.00 vol CO_2_, no antimicrobial) and **(B)** Chitosan addition (pH 5.0, 0.00 vol CO_2_, 0.2% chitosan product). *Limit of detection was <3 CFU/mL at day 1 (graphically represented as 2.9 CFU/mL) and <1 CFU/mL at days 7, 14, 28, and 60 (graphically represented as 0.9 CFU/mL).

In addition to the factorial treatment combinations, two alternative protection strategies were evaluated to represent approaches that fall outside the primary pH–antimicrobial–carbonation design. These treatments included kettle souring and chitosan addition, which were selected to examine whether other preservation approaches could provide microbial control in model non-alcoholic beer systems (Figure 4).

The kettle sour treatment (Figure 4A; Supplementary Tables S2–S7) showed the strongest bactericidal potential, reducing bacterial populations down to the initial limit of detection measured on day 1 (< 3 log CFU/mL) then down to the final limit of detection (< 1 log CFU/mL) at all other timepoints for *S.* Javiana, *L. monocytogenes*, *P. aeruginosa*, and *B. cereus*. One replicate resulted in recoverable *E. coli* colonies on day 7, but it remained below the limit of detection for the following sampling times.

The Chitosan blend (Figure 4B; Supplementary Tables S2–S7) did not show initial efficacy against *S.* Javiana. The count on day 60 remained at nearly 3 log CFU/mL. *E. coli* behaved similarly, with an initial drop in population on day 1, but recovered greater populations on day 7, before ultimately declining but recoverable on day 60. *L. monocytogenes*, *P. aeruginosa*, and *B. cereus* were initially inhibited and decreased in population to the limit of detection by day 14. However, *P. aeruginosa* showed significantly higher populations at timepoint 60 in one of its replicates (3.8 log CFU/mL vs. < 1 log CFU/mL).

### 3.4 Chemical analysis of NAB matrices

Basic beer chemistry analysis (°Plato, pH) was conducted after kegging but before full carbonation and inoculation. A single bottle of each treatment was also collected at day 60, autoclaved, and reanalyzed. A table of these results can be found in the supplementary information (Supplementary Table S8). Briefly, the kettle sour showed the lowest starting °Plato (5.59 vs. the target 6.00 [± 0.3] for all other treatments), representing the loss of nutrients consumed during the kettle souring process. Measurement of free amino nitrogen (FAN) also revealed a decrease between the 6 °P DME (204 ppm) and kettle sour (188 ppm) treatments. The °Plato increased between pre-inoculation and post-trial samples, likely due to the increase in biomass from the added bacterium. The measurement of °Plato is based on density, and while generally used as a proxy for fermentable sugars in beer, it is impacted by all soluble solids. The pH generally decreased for pH 5.0 treatments and increased for pH 4.2 treatments. However, as this measure was taken post-autoclaving, there may have been a shift in pH due to limited buffering capacity, resulting in a change in pH from the experimental conditions (Li et al., 2016; Leitzen et al., 2021).

## 4 Discussion

These results show that moderate carbonation levels (1.50 volumes) are vital in controlling *Salmonella* and *E. coli* in non-alcoholic beer systems. This affirms the conclusion of Rachon et al. (2024) that carbonation is inhibitory to their growth, although this prior work did not reach a conclusion on the critical value of CO₂ needed for inhibition. A follow-on study by Rachon et al. (2026), started in parallel with this research, has established that 2.4 g/L of CO₂ (1.22 vol) is enough to inhibit these bacteria in nutritionally depleted NAB (<0.01% sugar), but not when sugar was added (1.25%). A higher carbonation value of 3.8 g/L (1.94 vol) was able to inhibit growth in all the treatment conditions (Rachon et al., 2026). The 1.50 vol treatment tested in the presented study falls between those values and was sufficient to inhibit the growth of *S.* Javiana and *E. coli* O157:H7 across all pH and antimicrobial combinations in the 6 °P DME base. The results of the presented study and Rachon et al. are encouraging, as both show inhibition below typical commercial carbonation levels in packaged NAB, showing that when the beverage is properly carbonated, prior concerns about pathogen growth in uncarbonated NAB may be mediated (Azeredo et al., 2016; Çobo et al., 2023; Rachon et al., 2024).

Depending on the production method, NAB can vary substantially in residual nutrient composition. Products that are fully fermented and subsequently physically dealcoholized may contain lower residual nutrient levels, whereas biologically produced NABs often retain higher concentrations of residual sugars, such as maltose and maltotriose, as well as free amino nitrogen (FAN) (Quain, 2021; Rettberg et al., 2022; Britton and Hill, 2025; Myncke et al., 2025). The NAB matrices produced for this study were generally representative of commercially available biologically produced NABs with respect to residual extract (RE), despite not undergoing fermentation. While the FAN values measured in this trial were nearly twice those reported by Myncke et al. (2025) for a 6.5 °P original gravity wort, they still fell within the upper range of residual FAN values reported for commercial NABs (Lafontaine et al., 2020; Bauwens et al., 2021; Quain, 2021; Rettberg et al., 2022). While recent work by Rachon et al. (2026) found that added sugars supported growth at moderate carbonation, prior work by Witter et al. (1958) found that increasing concentrations of sucrose (0, 5, 10, and 15 °Brix) increased the death rate of *E. coli* in carbonated beverages. Further study should explore the role of nutrient modulation, such as FAN and RE, on lethality in combination with these presented intrinsic factors.

An important finding from these results is that not all bacteria are susceptible to carbonation, and at the tested levels in this study, *L. monocytogenes* was able to grow in the presence of 1.50 vol carbonation and the absence of other limiting hurdles. Effective control of *L. monocytogenes* required acidification to pH 4.2 or the inclusion of hop acids. This is consistent with prior work showing that hop compounds are an important hurdle in beer, particularly against Gram-positive organisms (Moir, 2000; Menz et al., 2009; Menz et al., 2010; Schurr et al., 2015). The marketing of these beverages to populations that should not consume alcohol overlaps with populations more susceptible to foodborne illness, such as pregnant women (Smith, 1999; Bowdring et al., 2024). Listeriosis is of particular concern for pregnant individuals because infection carries the risk of severe fetal outcomes, such as spontaneous abortion (Smith, 1999). Market surveys and recent reviews of commercial NAB show variability in bitterness and other microbial hurdles, indicating that these parameters should not be assumed without validation (Lafontaine et al., 2020; Quain, 2021; Rettberg et al., 2022; Rachon et al., 2024; Britton and Hill, 2025). It is critical to ensure that hops used meet effective inhibitory levels, particularly because some emerging aromatic hop products marketed toward improving NAB flavor do not contain the same hop acid content as traditional hop products (Moir, 2000; Schonberger et al., 2025). This is also a potential concern in products similar to NAB, such as non-alcoholic THC seltzers, which may not contain hops.

A potential protective effect for *S.* Javiana and *E. coli* in the presence of hop acids was also considered. Rachon et al. found that hop acid concentrations were not relevant for inactivation of *Salmonella Enteritidis* and *E. coli* O157: H7 across the tested levels of 10–50 BU, which is consistent with the limited activity of hop compounds against Gram-negative bacteria (Moir, 2000; Rachon et al., 2024). However, when comparing the no antimicrobial treatment to 10 ppm iso-*α*-acids (~10 BU) in the present study, the inactivation of these bacteria appears to be slower at the 1.50 vol carbonation levels when hops are present, across both pH 5.0 and 4.2 (Figures 2C vs. F, Figure 3C vs. F). This could be an effect of sublethal exposure to hop acids, leading to an adaptive response and increased protective measures such as proton pumping, although this mechanism was not directly tested in the present work (Usaga et al., 2014; Gavriil et al., 2024). However, this same effect is not observed in the 100 ppm potassium sorbate trials (Figures 2I, 3I), which would also be expected to operate through a weak-acid diffusion mechanism (Lambert and Statford, 1999; Stopforth et al., 2005). This suggests that the response may not be explained by weak-acid diffusion alone.

*Pseudomonas aeruginosa* and *B. cereus* were less successful than the other pathogens in the NAB matrices, which could be due to competition for resources or other intrinsic factors of the matrices that limited their potential, such as oxygen availability. Even in the non-carbonated samples, oxygen is limited, leaving aerobes at a disadvantage relative to facultative anaerobes. This is consistent with brewing microbiology literature indicating that low oxygen availability limits the relevance of many aerobic contaminants in finished beer (Menz et al., 2009; Quain, 2021; Britton and Hill, 2025). *P. aeruginosa* populations only mildly declined in the higher-pH treatment with no carbonation, especially in the chitosan blend treatment. It may be worth repeating trials for these two bacteria independently to remove the effects of competition, as there are potential vectors of concern for contamination of NAB through water and grain (Akond et al., 2009; Munford et al., 2017; Brar and Danyluk, 2018; Britton and Hill, 2025). Controlling pH appears to be an important critical value for the control of both organisms. For *B. cereus*, prior work has shown that beer-associated isolates can possess hop resistance and spoilage-associated traits, further supporting the need for organism-specific consideration in NAB systems (Wang et al., 2017).

The chitosan blend used in this study was broad-spectrum. It is important to note that specific molecular weights may be better to use to target certain bacteria (Hosseinnejad and Jafari, 2016). The commercial blend used in this study at the recommended dosage rate could not fully reduce each bacterial population to the limit of detection (3.8 log reduction). The lowest efficacy was observed for *S. Javiana* at < 2 log reduction, compared with the published target of 3–5 log reductions for this product. This highlights the importance of specific matrix effects and of conducting challenge studies for chemical preservation whenever there are substantive formulation changes or changes to the product risk matrix (National Advisory Committee on Microbiological Criteria for Foods, 2010). In addition, only a single hurdle was tested here, and this treatment should be explored further in additive combinatorial systems that present a more accurate matrix environment for NAB specifically.

Kettle souring was the most promising strategy evaluated in this study, showing a reduction to the limit of detection of all pathogens. This treatment is directly comparable to the 0.00 vol, no antimicrobial treatment at both pH 5.0 and 4.2, to more directly examine the impact of pH change. The pH of this matrix appears to be below the growth range of these pathogens and to induce bactericidal effects. This is notable considering acidified-food cold-fill-hold research has centered primarily around acetic acid, with some use of citric acid, but a lack of published data utilizing lactic acid (Breidt et al., 2018; Lobo et al., 2019). However, this trial was kettle-soured with *Lactiplantibacillus plantarum*, which likely also reduced available nutrients during the acidification process (Supplementary Table S8). This treatment should therefore be repeated with exogenous lactic acid addition to pH < 3.4 to verify whether the observed lethality is due to pH alone or to the broader effects of kettle souring. This may substantiate the greater exploration of lactic acid as an effective cold-fill-hold acidifying agent, especially in applications where acetic acid may be interpreted as an off-flavor defect. An acidification preservation strategy may also be particularly relevant for sour NAB styles, where acidification aligns with positive consumer attributes (Lafontaine et al., 2020).

Advancing the understanding of intrinsic hurdles is key to mapping the microbial risk in NAB for scenarios where thermal pasteurization strategies are unavailable or ineffective, falling outside of the safe harbor of current best practices. Recent work by Frierson et al. (2026) reported that *Salmonella* Tennessee persisted for at least 183 days in non-alcoholic beer under very low pasteurization conditions (0.75 PU), indicating that under-processing during thermal stabilization led to insufficient pathogen control in a NAB matrix lacking carbonation. In addition, Roselli et al. (2026) found that 54.7% of draught no- and low-alcohol beers sampled in the UK market were of unacceptable microbiological quality, and that poor quality was associated with higher gravity and higher pH, compounded by site-specific hygienic handling issues. Together, these findings further support that the presence of several intrinsic hurdles, such as pH reduction, carbonation, reduction of residual extract, and appropriate chemical stabilization, may enhance NAB safety in the case of under-processing or post-lethality contamination.

## 5 Conclusion

These results demonstrate that NAB can be formulated to be hostile toward common pathogenic bacteria, such as *S. Javiana*, *E. coli*, *L. monocytogenes*, *P. aeruginosa*, and *B. cereus*, thereby preventing growth under the conditions tested. The most robust protection was observed when multiple hurdles were combined, particularly low pH (≤ 4.2), moderate hop addition (10 BU), and moderate carbonation (≥ 1.50 vol). These parameters fall within a formulation space relevant to commercially produced and sensorially acceptable NAB, while also providing insight into the microbiological boundaries that may arise when one or more of these factors are absent or in related low- and no-alcohol beverage categories.

This study evaluated an initial inoculation level of 4.8 log CFU/mL for each bacterium, which is below the concentration needed to establish equivalency to a validated 5-log reduction thermal process. Additional work at higher inoculation levels is needed to determine whether these hurdle combinations could support replacement or reduction of pasteurization targets, particularly for draft applications and other higher-risk dispense systems. The use of a bacterial cocktail allowed for the testing of a greater number of bacterial species; however, it could have influenced the growth ability of certain strains due to resource competition, prompting a recommendation for further confirmation with single-strain trials. Additionally, data were only collected for 60 days, and follow-up studies may be needed to determine if viable but non-culturable bacteria could survive and present a threat in extended shelf-life settings. Nevertheless, these findings provide an important framework for designing NAB formulations with improved pathogen control and may help inform future strategies to reduce thermal intensity while still achieving microbiological safety.

## Data Availability

The original contributions presented in the study are included in the article/Supplementary material, further inquiries can be directed to the corresponding authors.
