# Supplementary material for: Effects of combinatorial hurdles on a non-alcoholic beer matrix challenged with Salmonella Javiana, Escherichia coli, Listeria monocytogenes, Pseudomonas aeruginosa, and Bacillus cereus
Source: Front Microbiol. 2026 May 18;17:1835393. doi: 10.3389/fmicb.2026.1835393 (PMC13223013; doi:10.3389/fmicb.2026.1835393)
Supplement: Supplementary file 1 [file Table_1.DOCX]

**Supporting Information**

**Effects of Combinatorial Hurdles on a Non-Alcoholic Beer Matrix Challenged With Salmonella Javiana, Escherichia coli, Listeria monocytogenes,**

**Pseudomonas aeruginosa, and Bacillus cereus**

Andrew Maust^1^, Karina Desiree^1^, Peter Rubinelli^1^, Sun Ferreira^1^, Jennifer Acuff^1*^, Scott Lafontaine^1*^

*^1^* *Department of Food Science, University of Arkansas, 2650 N. Young Ave., Fayetteville, AR, 72704, USA*

*Corresponding author(s) – Name: Scott Lafontaine Email: scottla@uark.edu

Name: Jennifer Acuff Email: jcacuff@uark.edu

**Table S1** – Treatment key for Tables S2-S8

| **Treatment #** | **Condition** |
| --- | --- |
| 1 | Kettle Sour |
| 2 | Chitosan |
| 3 | pH 5.0, 1.50 vol, no antimicrobial |
| 4 | pH 5.0, 0.75 vol, no antimicrobial |
| 5 | pH 5.0, 0.00 vol, no antimicrobial |
| 6 | pH 4.2, 1.50 vol, no antimicrobial |
| 7 | pH 4.2, 0.75 vol, no antimicrobial |
| 8 | pH 4.2, 0.00 vol, no antimicrobial |
| 9 | pH 5.0, 1.50 vol, 10 ppm iso-α-acids |
| 10 | pH 5.0, 0.75 vol, 10 ppm iso-α-acids |
| 11 | pH 5.0, 0.00 vol, 10 ppm iso-α-acids |
| 12 | pH 4.2, 1.50 vol, 10 ppm iso-α-acids |
| 13 | pH 4.2, 0.75 vol, 10 ppm iso-α-acids |
| 14 | pH 4.2, 0.00 vol, 10 ppm iso-α-acids |
| 15 | pH 5.0, 1.50 vol, 100 ppm potassium sorbate |
| 16 | pH 5.0, 0.75 vol, 100 ppm potassium sorbate |
| 17 | pH 5.0, 0.00 vol, 100 ppm potassium sorbate |
| 18 | pH 4.2, 1.50 vol, 100 ppm potassium sorbate |
| 19 | pH 4.2, 0.75 vol, 100 ppm potassium sorbate |
| 20 | pH 4.2, 0.00 vol, 100 ppm potassium sorbate |

|  | | | |  |  |  |  |  |  |  |  | |  | |  | |  |  |  |  | |  |  |  | |  |
| --- | --- | --- | --- | --- | --- | --- | --- | --- | --- | --- | --- | --- | --- | --- | --- | --- | --- | --- | --- | --- | --- | --- | --- | --- | --- | --- |
| **Treatment** | pH 5 Treatments | | | | | | | | | pH 4.2 Treatments | | | | | | | | | | | | | Alt Treatments | | | |
| **Timepoint** | 5 | 4 | 3 | 11 | 10 | 9 | 17 | 16 | 15 | 8 | 7 | 6 | | 14 | | 13 | | 12 | 20 | 19 | 18 | | 1 | | 2 |  |
| 0 | 4.80 AB | 4.80 B | 4.80 A | 4.80 B | 4.80 ABC | 4.80 A | 4.80 B | 4.80 A | 4.80 A | 4.80 C | 4.80 A | 4.80 A | | 4.80 BC | | 4.80 A | | 4.80 A | 4.80 A | 4.80 A | 4.80 A | | 4.80 A | | 4.80 A |  |
| 1 | 6.49 A | 4.86 B | 4.40 AB | 6.66 A | 6.06 A | 4.45 A | 6.63 A | 4.65 A | 4.25 A | 5.82 B | 4.62 A | 4.14 A | | 5.54 AB | | 4.32 A | | 4.02 AB | 3.92 B | 3.54 AB | 4.09 A | | 2.90 B | | 3.53 AB |  |
| 7 | 5.10 AB | 6.17 AB | 2.82 BC | 4.93 B | 5.31 AB | 2.75 A | 5.82 AB | 4.14 AB | 3.17 B | 6.89 A | 2.33 B | 3.53 A | | 6.18 A | | 1.90 BC | | 3.20 ABC | 0.93 C | 2.94 ABC | 2.20 B | | 0.90 C | | 3.99 AB |  |
| 14 | 3.57 B | 4.36 B | 2.48 CD | 3.07 C | 3.68 BC | 2.45 A | 5.26 B | 2.60 BC | 1.23 C | 4.17 C | 2.54 B | 1.47 B | | 3.90 C | | 0.90 C | | 2.85 BC | 0.90 C | 2.11 BC | 0.90 B | | 0.90 C | | 4.67 A |  |
| 28 | 0.90 C | 2.05 C | 2.26 CD | 1.03 D | 3.31 C | 2.52 A | 2.43 C | 2.36 BC | 0.90 C | 0.90 D | 4.59 A | 1.32 B | | 1.22 D | | 1.12 C | | 0.90 D | 0.90 C | 1.63 BC | 0.90 B | | 0.90 C | | 3.09 B |  |
| 60 | 0.90 C | 0.90 C | 0.90 D | 0.90 D | 0.90 D | 3.00 A | 1.97 C | 1.75 C | 0.90 C | 0.90 D | 2.85 B | 0.97 B | | 0.90 D | | 2.81 B | | 1.44 CD | 0.90 C | 0.90 C | 0.90 B | | 0.90 C | | 2.92 B |  |
| P-Value | 5.41E-06 | 1.44E-07 | 3.68E-05 | 5.68E-15 | 4.24E-06 | 0.0354 | 7.95E-08 | 0.000839 | 1.37E-09 | 6.27E-12 | 0.000153 | 4.41E-05 | | 4.39E-10 | | 1.38E-06 | | 7.41E-05 | <2e-16 | 0.00123 | 1.33E-05 | | <2e-16 | | 0.00128 |  |
| Significance | Yes | Yes | Yes | Yes | Yes | Yes | Yes | Yes | Yes | Yes | Yes | Yes | | Yes | | Yes | | Yes | Yes | Yes | Yes | | Yes | | Yes |  |

**Table S2**: *Salmonella* Javiana ANOVA Mean LOG CFU/ml

Means comparison groupings were generated using Tukey's Honestly Significant Difference (HSD). Limit of detection was <3.0 CFU/ml at day 1 (represented as 2.90 CFU/ml) and <1.0 CFU/ml at days 7, 14, 28, and 60 (represented as 0.90 CFU/ml)

**Table S3**: *E coli* O157:H7 ANOVA Mean LOG CFU/ml

| **Treatment** | pH 5 Treatments | | | | | | | | | pH 4.2 Treatments | | | | | | | | | Alt Treatments | |
| --- | --- | --- | --- | --- | --- | --- | --- | --- | --- | --- | --- | --- | --- | --- | --- | --- | --- | --- | --- | --- |
| **Timepoint** | 5 | 4 | 3 | 11 | 10 | 9 | 17 | 16 | 15 | 8 | 7 | 6 | 14 | 13 | 12 | 20 | 19 | 18 | 1 | 2 |
| 0 | 4.80 BC | 4.80 B | 4.80 A | 4.80 A | 4.80 C | 4.80 A | 4.80 C | 4.80 A | 4.80 A | 4.80 A | 4.80 A | 4.80 A | 4.80 A | 4.80 A | 4.80 A | 4.80 A | 4.80 A | 4.80 A | 4.80 A | 4.80 A |
| 1 | 6.23 A | 4.81 B | 4.40 A | 6.14 A | 5.74 B | 4.38 A | 6.14 B | 4.51 A | 4.15 AB | 4.47 AB | 4.38 A | 3.59 B | 4.38 AB | 4.40 A | 3.07 B | 4.19 A | 3.09 B | 3.59 B | 2.90 B | 2.90 BC |
| 7 | 5.80 AB | 6.31 A | 2.93 B | 4.05 AB | 6.74 A | 3.21 B | 6.71 A | 4.45 A | 3.47 B | 5.68 A | 1.90 B | 2.79 C | 4.45 AB | 1.90 B | 3.07 B | 1.51 B | 1.92 C | 1.43 C | 1.27 C | 3.40 B |
| 14 | 3.92 C | 5.86 A | 1.71 C | 3.23 AB | 4.17 C | 2.65 B | 4.80 C | 3.86 A | 1.84 C | 2.79 BC | 2.08 B | 0.90 D | 3.05 BC | 0.90 C | 2.33 C | 0.90 B | 0.90 D | 0.90 C | 0.90 C | 3.23 B |
| 28 | 1.20 D | 3.30 C | 0.90 C | 1.35 B | 2.61 D | 0.90 C | 2.07 D | 1.78 B | 0.90 C | 1.46 CD | 0.90 C | 0.90 D | 2.42 C | 0.90 C | 0.90 D | 0.90 B | 0.90 D | 0.90 C | 0.90 C | 1.75 CD |
| 60 | 0.90 D | 0.90 D | 0.90 C | 0.90 B | 1.46 E | 0.90 C | 0.90 E | 1.57 B | 0.90 C | 0.90 D | 0.97 C | 0.90 D | 0.90 D | 1.23 BC | 0.90 D | 0.90 B | 0.90 D | 0.90 C | 0.90 C | 1.25 D |
| P-Value | 4.31E-09 | 2.34E-10 | 9.18E-09 | 0.000998 | 2.46E-10 | 2.28E-08 | 4.69E-13 | 6.95E-07 | 2.94E-07 | 3.49E-06 | 1.97E-12 | 1.35E-12 | 7.31E-06 | 5.00E-10 | 6.30E-12 | 3.93E-10 | 6.32E-08 | 7.69E-10 | 1.08E-09 | 6.07E-06 |
| Significance | Yes | Yes | Yes | Yes | Yes | Yes | Yes | Yes | Yes | Yes | Yes | Yes | Yes | Yes | Yes | Yes | Yes | Yes | Yes | Yes |

Means comparison groupings were generated using Tukey's Honestly Significant Difference (HSD). Limit of detection was <3.0 CFU/ml at day 1 (represented as 2.90 CFU/ml) and <1.0 CFU/ml at days 7, 14, 28, and 60 (represented as 0.90 CFU/ml)

**Table S4**: *Listeria monocytogenes* ANOVA Mean LOG CFU/ml

| **Treatment** | pH 5 Treatments | | | | | | | | | pH 4.2 Treatments | | | | | | | | | Alt Treatments | |
| --- | --- | --- | --- | --- | --- | --- | --- | --- | --- | --- | --- | --- | --- | --- | --- | --- | --- | --- | --- | --- |
| **Timepoint** | 5 | 4 | 3 | 11 | 10 | 9 | 17 | 16 | 15 | 8 | 7 | 6 | 14 | 13 | 12 | 20 | 19 | 18 | 1 | 2 |
| 0 | 4.80 AB | 4.80 A | 4.80 B | 4.80 A | 4.80 A | 4.80 A | 4.80 B | 4.80 B | 4.80 A | 4.80 A | 4.80 A | 4.80 A | 4.80 A | 4.80 A | 4.80 A | 4.80 A | 4.80 A | 4.80 A | 4.80 A | 4.80 A |
| 1 | 4.70 AB | 4.39 A | 4.08 B | 4.16 A | 3.95 A | 2.93 B | 4.85 B | 3.62 B | 3.55 AB | 4.36 A | 4.17 B | 3.73 AB | 2.93 B | 2.90 B | 2.90 B | 4.20 B | 2.90 C | 3.56 B | 2.93 B | 3.75 B |
| 7 | 5.59 A | 2.96 AB | 4.46 B | 2.58 BC | 2.17 B | 1.90 C | 5.93 A | 5.25 AB | 2.95 ABC | 3.66 B | 1.90 C | 2.79 B | 1.39 C | 0.90 C | 0.90 C | 2.70 C | 3.10 B | 3.47 B | 0.90 C | 1.55 C |
| 14 | 4.47 B | 4.38 A | 6.53 A | 2.84 B | 2.06 B | 0.90 D | 3.77 C | 6.75 A | 1.17 C | 2.76 C | 0.90 D | 0.90 C | 0.90 D | 0.90 C | 0.90 C | 1.26 D | 0.90 D | 0.90 C | 0.90 C | 0.93 C |
| 28 | 2.36 C | 0.90 B | 6.23 A | 2.54 BC | 2.39 B | 0.90 D | 3.68 C | 3.94 B | 0.93 C | 0.90 D | 0.90 D | 0.90 C | 1.03 CD | 0.90 C | 0.90 C | 0.90 D | 0.90 D | 1.39 C | 0.90 C | 0.90 C |
| 60 | 2.66 C | 1.95 B | 2.96 C | 1.46 C | 1.81 B | 0.90 D | 3.09 C | 2.17 C | 2.02 BC | 1.03 D | 0.90 D | 0.90 C | 0.93 CD | 0.90 C | 0.90 C | 0.90 D | 0.90 D | 0.90 C | 0.90 C | 0.90 C |
| P-Value | 5.49E-07 | 0.000529 | 2.78E-08 | 6.85E-06 | 3.74E-05 | <2e-16 | 2.54E-07 | 6.70E-06 | 0.00073 | 8.32E-11 | 2.50E-15 | 2.31E-07 | 1.47E-11 | <2e-16 | <2e-16 | 3.01E-11 | 2.84E-16 | 3.77E-07 | <2e-16 | 9.42E-10 |
| Significance | Yes | Yes | Yes | Yes | Yes | Yes | Yes | Yes | Yes | Yes | Yes | Yes | Yes | Yes | Yes | Yes | Yes | Yes | Yes | Yes |

Means comparison groupings were generated using Tukey's Honestly Significant Difference (HSD). Limit of detection was <3.0 CFU/ml at day 1 (represented as 2.90 CFU/ml) and <1.0 CFU/ml at days 7, 14, 28, and 60 (represented as 0.90 CFU/ml)

**Table S5**: *Pseudomonas aeruginosa* ANOVA Mean LOG CFU/ml

| **Treatment** | pH 5 Treatments | | | | | | | | | pH 4.2 Treatments | | | | | | | | | Alt Treatments | |
| --- | --- | --- | --- | --- | --- | --- | --- | --- | --- | --- | --- | --- | --- | --- | --- | --- | --- | --- | --- | --- |
| **Timepoint** | 5 | 4 | 3 | 11 | 10 | 9 | 17 | 16 | 15 | 8 | 7 | 6 | 14 | 13 | 12 | 20 | 19 | 18 | 1 | 2 |
| 0 | 4.80 A | 4.80 A | 4.80 A | 4.80 A | 4.80 A | 4.80 A | 4.80 A | 4.80 A | 4.80 A | 4.80 A | 4.80 A | 4.80 A | 4.80 A | 4.80 A | 4.80 A | 4.80 A | 4.80 A | 4.80 A | 4.80 A | 4.80 A |
| 1 | 4.31 A | 3.94 A | 3.75 B | 3.99 A | 3.95 B | 3.66 B | 4.49 AB | 3.73 B | 3.40 B | 3.30 B | 3.72 B | 3.33 B | 3.58 B | 3.23 B | 2.93 B | 3.26 B | 2.90 B | 2.97 B | 2.90 B | 2.90 AB |
| 7 | 3.72 A | 2.50 B | 2.07 C | 3.41 A | 1.90 C | 1.90 C | 3.34 AB | 1.92 C | 0.90 C | 1.93 C | 1.90 C | 1.90 C | 0.90 C | 0.90 C | 0.90 C | 0.90 C | 0.90 C | 0.90 C | 0.90 C | 0.90 C |
| 14 | 2.95 A | 0.90 C | 0.90 D | 1.27 B | 0.90 D | 0.90 D | 2.99 B | 0.90 D | 0.90 C | 0.90 D | 0.90 D | 0.90 D | 0.90 C | 0.90 C | 0.90 C | 0.90 C | 0.90 C | 0.90 C | 0.90 C | 0.90 C |
| 28 | 0.90 A | 0.90 C | 0.90 D | 1.27 B | 0.90 D | 0.90 D | 2.79 B | 0.90 D | 0.90 C | 0.90 D | 0.90 D | 0.90 D | 0.90 C | 0.90 C | 0.90 C | 0.90 C | 0.90 C | 0.90 C | 0.90 C | 0.90 C |
| 60 | 0.90 A | 0.90 C | 0.90 D | 0.90 B | 0.90 D | 0.90 D | 0.90 C | 0.90 D | 0.90 C | 0.90 D | 0.90 D | 0.90 D | 0.90 C | 0.90 C | 0.90 C | 0.90 C | 0.90 C | 0.90 C | 0.90 C | 1.88 BC |
| P-Value | 0.0248 | 5.14E-09 | 1.41E-11 | 1.24E-05 | 2.51E-13 | 2.86E-10 | 7.92E-05 | 1.79E-08 | 5.58E-09 | 1.71E-13 | 1.59E-13 | 8.15E-13 | 1.72E-14 | 6.93E-14 | <2e-16 | 5.86E-15 | <2e-16 | <2e-16 | <2e-16 | 7.46E-05 |
| Significance | Yes | Yes | Yes | Yes | Yes | Yes | Yes | Yes | Yes | Yes | Yes | Yes | Yes | Yes | Yes | Yes | Yes | Yes | Yes | Yes |

Means comparison groupings were generated using Tukey's Honestly Significant Difference (HSD). Limit of detection was <3.0 CFU/ml at day 1 (represented as 2.90 CFU/ml) and <1.0 CFU/ml at days 7, 14, 28, and 60 (represented as 0.90 CFU/ml)

**Table S6**: *Bacillus cereus* ANOVA Mean LOG CFU/ml

| **Treatment** | pH 5 Treatments | | | | | | | | | pH 4.2 Treatments | | | | | | | | | Alt Treatments | |
| --- | --- | --- | --- | --- | --- | --- | --- | --- | --- | --- | --- | --- | --- | --- | --- | --- | --- | --- | --- | --- |
| **Timepoint** | 5 | 4 | 3 | 11 | 10 | 9 | 17 | 16 | 15 | 8 | 7 | 6 | 14 | 13 | 12 | 20 | 19 | 18 | 1 | 2 |
| 0 | 4.80 A | 4.80 A | 4.80 A | 4.80 A | 4.80 A | 4.80 A | 4.80 A | 4.80 A | 4.80 A | 4.80 A | 4.80 A | 4.80 A | 4.80 A | 4.80 A | 4.80 A | 4.80 A | 4.80 A | 4.80 A | 4.80 A | 4.80 A |
| 1 | 2.97 B | 2.90 B | 2.90 B | 2.90 B | 2.90 B | 2.90 B | 2.97 B | 2.90 B | 2.90 B | 2.90 B | 2.90 B | 2.90 B | 2.90 B | 2.90 B | 2.90 B | 2.90 B | 2.90 B | 2.90 B | 2.90 B | 2.90 B |
| 7 | 0.90 C | 0.90 C | 0.90 C | 0.90 C | 0.90 C | 0.90 C | 0.90 C | 0.90 C | 0.90 C | 0.90 C | 0.90 C | 0.90 C | 0.90 C | 0.90 C | 0.90 C | 0.90 C | 0.90 C | 0.90 C | 0.90 C | 0.90 C |
| 14 | 0.90 C | 0.90 C | 0.90 C | 0.90 C | 0.90 C | 0.90 C | 0.90 C | 0.90 C | 0.90 C | 0.90 C | 0.90 C | 0.90 C | 0.90 C | 0.90 C | 0.90 C | 0.90 C | 0.90 C | 0.90 C | 0.90 C | 0.90 C |
| 28 | 0.90 C | 0.90 C | 0.90 C | 0.90 C | 0.90 C | 0.90 C | 0.90 C | 0.90 C | 0.90 C | 0.90 C | 0.90 C | 0.90 C | 0.90 C | 0.90 C | 0.90 C | 0.90 C | 0.90 C | 0.90 C | 0.90 C | 0.90 C |
| 60 | 0.90 C | 0.90 C | 0.90 C | 0.90 C | 0.90 C | 0.90 C | 0.90 C | 0.90 C | 0.90 C | 0.90 C | 0.90 C | 0.90 C | 0.90 C | 0.90 C | 0.90 C | 0.90 C | 0.90 C | 0.90 C | 0.90 C | 0.90 C |
| P-Value | <2e-16 | <2e-16 | <2e-16 | <2e-16 | <2e-16 | <2e-16 | <2e-16 | <2e-16 | <2e-16 | <2e-16 | <2e-16 | <2e-16 | <2e-16 | <2e-16 | <2e-16 | <2e-16 | <2e-16 | <2e-16 | <2e-16 | <2e-16 |
| Significance | Yes | Yes | Yes | Yes | Yes | Yes | Yes | Yes | Yes | Yes | Yes | Yes | Yes | Yes | Yes | Yes | Yes | Yes | Yes | Yes |

Means comparison groupings were generated using Tukey's Honestly Significant Difference (HSD). Limit of detection was <3.0 CFU/ml at day 1 (represented as 2.90 CFU/ml) and <1.0 CFU/ml at days 7, 14, 28, and 60 (represented as 0.90 CFU/ml)

**Table S7**: Total Bacterial Count ANOVA Mean LOG CFU/ml

| **Treatment** | pH 5 Treatments | | | | | | | | | pH 4.2 Treatments | | | | | | | | | Alt Treatments | |
| --- | --- | --- | --- | --- | --- | --- | --- | --- | --- | --- | --- | --- | --- | --- | --- | --- | --- | --- | --- | --- |
| **Timepoint** | 5 | 4 | 3 | 11 | 10 | 9 | 17 | 16 | 15 | 8 | 7 | 6 | 14 | 13 | 12 | 20 | 19 | 18 | 1 | 2 |
| 0 | 5.50 AB | 5.50 B | 5.50 B | 5.50 B | 5.50 B | 5.50 A | 5.50 B | 5.50 AB | 5.50 A | 5.50 B | 5.50 A | 5.50 A | 5.50 A | 5.50 A | 5.50 A | 5.50 A | 5.50 A | 5.50 A | 5.50 A | 5.50 A |
| 1 | 6.70 A | 5.28 B | 4.85 BC | 6.81 A | 6.25 AB | 4.83 AB | 6.77 A | 5.02 B | 4.73 A | 5.86 B | 4.96 A | 4.51 AB | 5.63 A | 4.71 A | 4.19 B | 4.63 B | 4.01 B | 4.46 B | 3.61 B | 4.09 BC |
| 7 | 6.47 A | 6.56 A | 4.50 C | 5.53 B | 6.85 A | 3.40 AB | 6.82 A | 5.35 AB | 3.78 AB | 6.93 A | 2.69 B | 3.95 B | 6.20 A | 2.26 BC | 3.56 BC | 2.81 C | 3.52 BC | 3.62 C | 1.77 C | 4.12 BC |
| 14 | 4.97 B | 5.89 AB | 6.53 A | 3.56 C | 4.33 C | 2.90 B | 5.45 B | 6.75 A | 2.19 BC | 4.22 C | 2.70 B | 1.95 C | 4.01 B | 1.60 C | 3.09 C | 1.71 D | 2.36 CD | 1.60 D | 1.60 C | 4.69 AB |
| 28 | 2.51 C | 3.35 C | 6.23 A | 2.72 D | 3.44 D | 2.83 B | 3.88 C | 3.96 BC | 1.61 C | 1.84 D | 4.59 A | 1.81 C | 2.55 C | 1.70 C | 1.92 D | 1.60 D | 1.94 D | 1.88 D | 1.60 C | 3.12 C |
| 60 | 2.69 C | 2.21 D | 2.97 D | 1.79 E | 2.19 E | 3.01 B | 3.23 D | 2.43 C | 2.48 BC | 1.64 D | 2.87 B | 1.61 C | 1.61 D | 2.90 B | 1.60 D | 1.60 D | 1.60 D | 1.60 D | 1.60 C | 3.09 C |
| P-Value | 1.32E-07 | 4.22E-08 | 9.23E-09 | 1.39E-10 | 3.04E-09 | 0.00647 | 5.07E-10 | 4.78E-05 | 6.67E-05 | 1.02E-10 | 4.09E-05 | 2.27E-07 | 4.60E-09 | 1.32E-07 | 3.99E-07 | 9.32E-14 | 8.24E-06 | 2.63E-09 | 1.36E-13 | 0.000297 |
| Significance | Yes | Yes | Yes | Yes | Yes | Yes | Yes | Yes | Yes | Yes | Yes | Yes | Yes | Yes | Yes | Yes | Yes | Yes | Yes | Yes |

Means comparison groupings were generated using Tukey's Honestly Significant Difference (HSD). Limit of detection was <3.0 CFU/ml at day 1 (represented as 2.90 CFU/ml) and <1.0 CFU/ml at days 7, 14, 28, and 60 (represented as 0.90 CFU/ml)

**Table S8:** Chemical Characterization of NAB Samples Before and After Inoculation

| Treatment | Pre-Inoculation | | Day 60 Post-Inoculation* | |
| --- | --- | --- | --- | --- |
|  | °Plato (Real Extract) | pH | °Plato (Real Extract) | pH |
| 1 | 5.59 | 3.39 | 5.64 | 3.37 |
| 2 | 6.00 | 5.01 | 6.39 | 4.13 |
| 3 | 6.00 | 5.01 | 6.30 | 4.41 |
| 4 | 6.00 | 5.01 | 6.19 | 4.54 |
| 5 | 6.00 | 5.01 | 6.02 | 4.41 |
| 6 | 6.00 | 4.20 | 6.43 | 4.26 |
| 7 | 6.00 | 4.20 | 6.40 | 4.46 |
| 8 | 6.00 | 4.20 | 6.27 | 4.33 |
| 9 | 6.30 | 5.03 | 6.58 | 5.02 |
| 10 | 6.30 | 5.03 | 6.44 | 4.57 |
| 11 | 6.30 | 5.03 | 6.36 | 4.50 |
| 12 | 6.30 | 4.23 | 6.60 | 4.32 |
| 13 | 6.30 | 4.23 | 6.63 | 4.36 |
| 14 | 6.30 | 4.23 | 6.46 | 4.36 |
| 15 | 6.10 | 4.97 | 6.39 | 5.09 |
| 16 | 6.10 | 4.97 | 6.35 | 4.46 |
| 17 | 6.10 | 4.97 | 6.27 | 4.43 |
| 18 | 6.10 | 4.23 | 6.53 | 4.27 |
| 19 | 6.10 | 4.23 | 6.46 | 4.27 |
| 20 | 6.10 | 4.23 | 6.47 | 4.21 |

*Post-Inoculation samples were autoclaved before being analyzed
